# The Effect of Processing on Digestion of Legume Proteins

**DOI:** 10.3390/foods8060224

**Published:** 2019-06-24

**Authors:** Donata Drulyte, Vibeke Orlien

**Affiliations:** Department of Food Science, Faculty of Science, University of Copenhagen, Rolighedsvej 26, DK-1958 Frederiksberg C, Denmark; wnb429@alumni.ku.dk

**Keywords:** legume protein, processing, digestibility

## Abstract

The domestic processing methods, soaking, cooking (traditional, microwave, pressure), and baking and the industrial processing, autoclaving, baking, and extrusion are used to improve consumption of legumes. The growing awareness of both health and sustainability turns the focus on protein (bio)availability. This paper reports the effect of these processing methods on the legume protein digestibility. Overall, the protein digestibility increases after processing by the different methods. However, since both the type of legume and the applied methods differ it cannot be concluded which specific method is best for the individual legume type.

## 1. Introduction

In today’s health-based consumer-focused world, more and more research is being conducted in order to obtain knowledge about the effects of animal- and plant-based diets on our health. It has generally been accepted that the consumption of meat can increase the incidence and prevalence of obesity, cardiovascular diseases and stroke, cancer, type 2 diabetes, and patient mortality. Contrarily, the plant-based diet has been accepted to lower the risk of these factors [1,2]. Consequently, an increasing percentage of people are changing their diets to become vegetarians, vegans, or flexitarians in order to lower their meat consumption and increase the amount of vegetables and fruits [3,4]. Yet, false information and health trends, which state that plant-based proteins are worse for your health, inadequate for building muscle, or are not proteinaceous enough, have become powerful and generally accepted misconceptions among some members of the population [5]. Research has managed to prove that legumes can be a good source of protein, though the bioavailability of animal proteins was still proven to be higher [6,7]. The recommended acceptable intake of proteins is around 0.8 g/kg for adults (defined as the average daily level of intake sufficient to meet the nutrient requirements of nearly all healthy people). Legumes have a great potential for delivering quality proteins, but raw legumes have a lower degree of bioavailability and, thereby, lower nutritional value as compared to other foodstuffs. Upon ending the last century, food was used to improve health, while entering the millennium a paradigm shift resulted in that our knowledge is now being used to improve foods in respect of healthiness. Both animal- and plant-based proteins have been widely investigated in order to understand the digestibility as such. Since it is well known that different processing procedures will affect both structure and functionality of proteins, it is anticipated that processing may also affect protein digestibility. This communication surveys the effect of different processing methods on legume protein digestion (PD) in order to contribute to the knowledge of protein digestibility. It is not a comprehensive review of all research studies on PD, but is based on the studies defined by investigations that contribute to provide an overview of the effect of processing on digestion of legume proteins. Thus, to “set the current scene” and give an outline of the challenges and future perspectives.

## 2. Processing Techniques 

Consumption of raw legumes can be tedious and difficult, and, in worst case, inefficient with respect to amino acid absorption in the gut. Empirical, various domestic processing methods are used to ease the legume consumption per se, but without grasping the effect on protein digestibility as such. Several different types of legumes and of processing techniques have, thus, been investigated in order to evaluate the potential improvement of nutritional value and protein utilization. Processing is the action of performing a series of mechanical or thermal operations on food in order to change or preserve it. It may involve soaking, cooking, microwave irradiation, baking, pressure-cooking, autoclaving, and extrusion [8,9]. Since the processing methods are performed at different conditions, a brief description of the techniques that have been used is presented. 

One of the commonly used pre-processing method is soaking. The most important parameters of soaking are: product:water ratio, temperature of the soaking water, and the duration. These parameters differ considerably depending on the type of legume, hence, the legumes are soaked in either hot or boiling tap or distilled water, with legume:water ratios varying from 1:1 to 1:5, and soaking durations ranging from 12 to 16 h. Traditional cooking is the simplest thermal processing method used. In the case of legumes, they are usually either placed in surplus warm water and slowly brought to boiling or directly put into boiling water (100 °C) in a pot. The time of the cooking process is a very important factor, which can differ depending on the legume type and if the legume seeds were soaked, dried, or not treated prior to cooking. Nevertheless, in a majority of the reviewed studies, the cooking time varied between 20 and 40 min. Microwave radiation is a more intense and, thereby, faster thermal method than conventional cooking. The thermal effects are related to the heat generated by the absorption of microwave energy by the water in the food matrix. Contrarily to cooking, microwave processing offers the opportunity to provide a more precise amount of energy to the food item. In addition, the heating effect of microwave can be tuned (energy dosage) and is considerably faster, almost immediate, resulting in reduction of cooking time. Similar to cooking, the microwave treatment time varies depending on type of legume and pre-treatment before microwave cooking. The capacity of microwave ovens differs due to different microwave frequency, thus delivering different energy doses, which will influence the treatment duration. In the studies herein, the doses of energy varies from 500 to 2000 J/g, usually being increased by 250 J/g. Pressure-cooking and autoclaving are two other high-intensity thermal methods. They are both based on exceeding normal, ambient boiling point of water in a sealed vessel (pressure cooker) or a pressure chamber (autoclave). The pressure cooker works by trapping the steam produced from boiling water of 121 °C inside the vessel. Many autoclaves work by subjecting the item to pressurized saturated steam at 121 °C. Thus, they both operate at 1.8 to 2.0 bar in order to obtain this temperature. The evaluated temperature and pressure generally increase reaction rates, thus the duration of pressure cooking and autoclaving is less than traditional cooking, though it varies between the two. Pressure cooking is usually performed in 5 to 15 min, whereas autoclave time may vary from seven to 60 min, both depending on the type and quantity of legume. The last two processing methods are based on milling the legumes into a flour prior to treatment. Baking is an old, traditional thermal method that uses dry air at ambient pressure to apply heat. Generally, a dough based on legume flour is made, rested for some time (leavening), and then baked at temperatures varying from about 180 to 220 °C for more than 30 min. Extrusion is also based on transforming legume flour into an edible food product. The extrudates are prepared using either a single- or twin-screw extruder that works under high temperature and high mechanical pressure. The main independent parameters, such as particle size of flour, feed rate, moisture content, barrel temperature, and screw speed, during the extrusion process, differ a lot depending on legume type and extruder parameters (type of mill, screen hole size, etc.). In the reviewed papers, moisture content was about 22–25%, while temperature and screw rotation speed varied considerably from 30 to 150 °C and from 100 to 650 rpm, respectively. 

## 3. Protein Digestibility Methodologies

The major difference between PD assessment is if the measurement is conducted in vivo or in vitro. Furthermore, the digestion of proteins may be determined by a broad variety of methods within these two PD categories. In vivo methods are based on feeding trails using animals or humans. The most accurate result is obtained with controlled feeding experiments with animals, usually rodents, chicken, or pigs are used. However, care should be taken upon transferring results and conclusions from animal tests to human, since the human GI tract differs considerably from animals. On the one hand, human intervention studies are the golden method for assessing protein digestion and nutritional value. On the other hand, in vivo experiments are time consuming and costly, and difficult to control if the study is based on human intervention. Therefore, in vitro models simulating the human GI tract have been used. Contrarily to in vivo methods, in vitro methods are easier to control, rapid to conduct, and may be less expensive. The drawbacks among the in vitro methods are differences in the operation and the experimental parameters; as a result, there are considerable differences in the measured digestibility complicating comparison of results. In addition, the difficulties in accurately transferring and simulating the complex digestion process in the human GI tract complicates the performance of these laboratory experiments and caution must be taken upon interpretation of results. In the following, the various methods used to measure and assess legume protein digestibility in the surveyed papers are summarized.

### 3.1. In Vitro Protein Digestibility (IVPD)

The in vitro protein digestibility assay is the most used method for analyzing digestibility of protein samples. The analysis is carried out by first preparing a multi-enzyme solution, usually including trypsin, chymotrypsin, and peptidase, with some exceptions using single trypsin [10] or pepsin solution [11], or, sequentially, pepsin and pancreatin solutions [12,13,14]. Commonly, an aliquot of the enzyme solution is added to a solution of the sample at pH 8.0. The mixture of sample and enzyme solution is incubated for 10 min at 37 °C. The pH decrease during the incubation period is recorded, followed by calculations of the IVPD. The pH decrease is caused by the free amino acid carboxyl groups from the protein chain released by the proteolytic enzymes during the digestion.

### 3.2. Crude Protein (CP)

Crude protein is the quantity of proteins in a specific sample of feed or food. The crude protein is determined from the total content of nitrogen obtained by a total degradation of the proteins. The crude protein percentage is calculated using the Kjeldahl conversion factor of 6.25 [15,16].

### 3.3. Ileal Digestability (AID, SID)

The ileal digestibility of the proteins under investigation is based on intervention study. Thus, different protein diets (usually with similar crude protein and trypsin inhibitor activity levels) are fed to animals, usually rats, pigs, or chickens, living under controlled conditions. The feeding trials may run from 10 to 20 days depending on study and animal. Afterwards, the animals are euthanized and the ileal digesta is collected by mildly flushing the digesta with clean distilled water and the amount of crude protein or amino acids is measured. Overall, the ileal protein digestibility is the difference between the amount of protein or amino acid ingested by the animal and the amount of protein or amino acid in the ileal digesta outflow. However, the calculation of the ileal digestibility depends on which amount of the ileal protein/amino acid outflow is used in the actual calculation and can be calculated as apparent (AID), true (TID), or standardized (SID) ileal digestibility. The AID for a certain amino acid is computed by subtracting the total ileal outflow of that specific amino acid from the quantity consumed by the animal. The values for SID are computed similarly to the values for AID, apart from the fact that the basal ileal endogenous amino acid (AA) losses (IAAend) are subtracted from the ileal outflow [17,18,19].

## 4. Effect of Processing on Protein Digestibility

Legumes are rich sources of proteins (18–41%) and are important raw food materials worldwide. The proteins provide the essential amino acids necessary for maintaining body muscle and growth. However, legumes, and, thereby their proteins, are not similar. Moreover, the nutritional quality of proteins is not solely dictated by the AA composition, but important factors like digestion rate and digestibility in the GI tract, which in turn are determined by the protein structure and enzyme accessibility, are of outmost importance. The protein digestibility is affected by both endogenous and exogenous factors. Endogenous factors relate to the protein as such, that is, protein structural characteristics and how and to what extent food processing may affect this structure. Exogenous factors are related to the food matrix, and include protein interactions with other compounds like carbohydrates, lipids, and especially anti-nutritional factors (ANF). ANFs include the anti-nutritional proteins like trypsin inhibitors and lectins and the anti-nutritional chemicals like tannins, phytates, and polyphenols. Therefore, it may beforehand be expected that both different types of legume protein and different processing may result in a very diverse digestibility. Indeed, Table S1 (Supplementary Materials) shows the difference in protein digestibility of legumes using various processing methods.

### 4.1. Cooking

The simplest processing technique, conventional cooking, is the most studied method with 12 studies as seen in Table S1. Four different types of legumes, beans, peas, lentils, and chickpeas, but of various cultivars, were investigated. Most of the investigations were reporting in vitro protein digestibility, while only few studies reported protein content changes [15] and in vivo digestion [16,20]. The protein content in the flour of unprocessed bean, pea, and lentil is rather similar, ranging from 24.8% to 28.7% [15]. However, comparing legume subtypes that underwent cooking, the effect on the protein content differed considerably (Table S1). Unprocessed bean (*Phaseolus vulgaris*) with protein contents of 24.8% (Raba) and 26.2% (Warta) significantly decreased to 23.0% and 21.3%, respectively. The protein content in cooked and uncooked peas (*Pisum Savitum*) yielded quite different results, since the content in the Milwa cultivar increased, while the Medal’s protein content decreased [15]. The lentil cultivars (*Lens culinaris*) demonstrated both the highest and a significant increase in protein content after cooking. However, digestibility was not investigated [15]. For all cooked legumes, IVPD increased significantly, as seen in Table S1. For the flour of eight different species of unprocessed peas (*Pisum sativum* L.) the IVPD ranged from 79.9% to 83.5%, while the IVPD of the processed samples varied between 85.9% and 86.8% [21]. The IVPD of 83.61% for unprocessed chickpea (*Cicer arietinum* L.) increased to 88.52% after cooking for 90 min [22]. The time of cooking was found to be important for the IVPD, since Habiba et al. [23] found an increasing IVPD upon increasing cooking time. Interestingly, the increased IVPD was concomitant with a decrease in total protein content. This decrease in total crude proteins was suggested to be a result of leaching of water-soluble proteins during cooking [23]. A similar explanation is likely for the reported decreases in protein content for some of the legumes mentioned above [15]. The cooking resulted in improved IVPD of lentils, chickpea, peas, and soybean, but soaking the legumes prior to heating did not result in consistent significant effects [24]. Similarly, cooking of three different varieties of kidney beans significantly increased the IVPDs, while pre-soaking did not have any major effect [25]. Moreover, soaking in alkaline solution (sodium bicarbonate, pH 8.2) did not improve protein digestibility. However, Embaby [26] found that soaking cooked bitter and sweet lupin seeds for 96 and 24 h, respectively, further improved the IVPD. 

In same line, the in vivo digestibility differed according to the lentil type, temperature, and time applied. Digestibility (SID) of unprocessed full-fat soybeans (FFSB) was 46% [16]. Incremental increase of the cooking temperature and duration caused a correlated increase in the soybean’s SID. Hence, beans cooked at 80 °C for one min had a SID of 52%, whereas FFSB processed at 100 °C for six or 16 min had a SID of 73% and 80%, respectively, Table S1. Similarly, the cooking of peas (*Pisum sativum* L.) prior using as a diet resulted in an increase of true digestibility (TD) (79.8%) as compared to raw pea diet with TD of 74.7% [20]. 

For centuries, prior human consumption legume seeds have been soaked and thermally treated by conventional cooking due to the simplicity in the execution and equipment. However, the drawbacks of cooking are a fairly uncontrolled and non-adjustable process and the potential loss of valuable nutrients like vitamins. Therefore, other processing techniques are investigated in order to optimize the protein digestion by better control of the heating process.

### 4.2. Microwave Cooking

IVPD of three faba bean cultivars before processing were 46.0%, 52.2%, 51.5% for Windsor White, Bacchus, and Basta, respectively, and, thereby, characterized by a markedly lower IVPD compared to other varieties of seeds reported in former section [27]. Generally, treatment with microwave radiation resulted in an increase in protein digestibility of all bean types (Table S1). The lowest amount of energy (500 J/g) caused a significant increase in protein digestibility from 46.0%, 52.2%, and 51.5% to 57.1%, 68.0%, and 53.2%, respectively [27]. Further increase in energy to 1000 J/g significantly improved protein digestibility to 76.5% for Windsor White, 76.1% for Bacchus, and 78.2% for Basta. However, more energy input (1250, 1500, 1750 J/g) during microwave cooking did not significantly affect the protein digestibility further [27]. The authors concluded that microwave processing at 1000 J/g is optimal for the protein digestibility of faba beans [27]. Soaking is a traditional domestic method for preparing seeds for further processing. Embaby [26] investigated the reverse situation, thus soaking of bitter and sweet lupin seeds for 96 and 24 h, respectively, after microwave treatment. It was found that microwave processing significantly improved the IVPD by 2.5% and 1.5% compared to the raw seeds (from 78.55% to 80.40% for bitter lupin and from 79.46% to 80.67% for sweet lupin, Table S1). Thus, soaking following microwave cooking further improved the IVPD for bitter lupin seeds, while no significant increase was found for the sweet lupin seeds [26]. It is noted that the considerably longer soaking time (96 h) for the bitter lupin seeds compared to 24 h for the sweet lupin seeds may have a major impact on this observation, though the author did not comment on this. 

### 4.3. Pressure Cooking

Pressure cooking is another common domestic treatment method utilizing the high-energy input to shorten processing time. Protein digestibility of four unprocessed moth bean cultivars differed significantly between 70.3–74.6% for the local variety to new varieties [12]. After pressure-cooking, IVPD increased to around 78% for the local, Jwala, and RMO 225, while RMO 257, with the highest raw IVPD, also had the highest cooked IVPD of 82.4% (Table S1). Soaking prior pressure cooking in addition to reducing processing time, positively affected bean protein digestibility, since the IVPDs were improved by 14–16% [12]. Pressure cooking of peas was also found to improve the digestibility of proteins compared to digestibility of raw peas [23]. The IVPDs resulting from standard pressure cooking were at the same level as the IVPDs obtained after cooking irrespective of processing times, though a shorter treatment time was necessary for pressure cooking, while slightly higher than the IVPDs after microwave treatment (Table S1) [23].

### 4.4. Autoclaving

Autoclaving of beans and peas significantly reduced the content of crude proteins compared to the raw legumes, while the content in the lentils was unaffected (Table S1) [15]. Hence, the autoclaving treatment of raw legumes had different effect on the protein content compared to the cooking process. Small differences between the two heat treatments were also observed for the Milwa pea and the two lentil cultivars [15]. The two studies reporting the effect of autoclaving of yellow peas [28] and soybean [15] on in vivo protein digestibility showed an increasing tendency, Table S1. Though the legume types, the processing conditions, and the animal model differed, it seems that the rather harsh heat treatment of the plant material prior inclusion in the diet improved the protein digestion in the GI tract. Similarly, autoclaving generally increased the IVPD significantly for all types of legumes investigated, expect for faba bean and lentil [29] (Table S1). Hence, unambiguous improvement of protein digestibility was not obtained after autoclaving compared to cooking. The more harsh and intense heat treatment by autoclaving did seemingly not always have a positive effect. Furthermore, the increase of autoclaving time from 10 to 90 min significantly reduced the IVPD of four different legumes [11]. 

### 4.5. Baking

House and co-workers have investigated the effect of baking of pea and lentil flours on protein digestibility [30,31]. Apparently, the process of mixing, kneading, rising, and baking of the dough reduced the IVPD compared to cooking, except for the red lentil (Table S1). Thus, the authors concluded that, for home preparation of these legumes, cooking is more advantageous than baking [31]. 

### 4.6. Extrusion

Extrusion is a thermal process with high energy efficiency due to high shear and compression, and probably the most severe thermal treatment method. Nevertheless, the extrusion process had a positive effect on the nutritional value of legumes. Extruding the flour of common beans, pea seed, faba, and kidney beans significantly increased the IVPD up to 87% [14,32,33]. The in vitro digestibility results were supported by in vivo feeding experiments. Hence, feeding chicken with extruded peas or kidney beans improved the effect on the apparent ileal digestibility of crude protein [34,35]. The AIDs of CP for unprocessed and extruded pea seeds (*Pisum sativum L., Tarachalska cv*.) were 74.3% and 85.9%, respectively, thus extrusion increased protein digestibility [21]. Inclusion of extruded kidney bean (100–300 g/kg) in broiler diets increased AID to 85.5–85.9% compared to AID of 77.23–79.03% with feed based on raw kidney beans [35]. 

### 4.7. New Processing Methods

High pressure and ultrasound are non-thermal technologies known to only affect non-covalent interactions in macromolecules. Therefore, the possible pressure-induced protein unfolding may enhance the access of the digestive enzymes to the cleavage sites in the protein, thus improving digestibility. However, as seen in Table S1, the effects on IVPD of lentils, chickpeas, peas, and soybean after high pressure or ultrasound treatment were inconsistent or insignificant compared to the protein digestibility of the raw legumes [10,24].

### 4.8. Factors Affecting PD

Various processing techniques affect legume PD to various extents compared to the PD of raw legumes. Generally, the IVPD increases after processing by different heating methods. The improvement in PD was attributed to protein denaturation. Thus, the increased digestibility was ascribed to the resulting heat-induced denaturation of the proteins, thereby enhancing accessibility of susceptible sites to proteolysis. On the other hand, digestibility can be compromised by protein aggregation due to the thermal treatment [29]. A consequence of protein denaturation is increased opportunity for various intra- and intermolecular interactions, especially disulfide bridges between amino acids containing free thiol groups. Crosslinked, aggregated proteins are less accessible to digestive enzymes because of a different localization of amino acid residues specific for protease action resulting in lack of PD improvement. It is noted that the degree of amino acid reactivity and extent of aggregation differs under various conditions and not all amino acids participate in protein crosslinking irrespective of the processing condition. In addition, the native protein conformation of the primary legume proteins, globulins and albumins, may also affect the PD as such. The albumins have a compact globular structure stabilized by a large number of disulfide bonds, thus possessing an inherent structural hindrance limiting enzyme access [21]. In that respect, it was observed that an increased proportion of globulins and decreased proportion of albumins in pea seeds could contribute to an increased IVPD [21]. This may indicate that the ratio of albumin:globulin also has an influence on the IVPD of legumes. It is generally accepted that the abundance of ANF in plant protein sources contributes to the lower digestibility compared to typical mammal proteins. Phytic acid, tannins, and polyphenols may interact with protein to form complexes by cross-linking with the proteins, resulting in a decreased protein solubility and making these protein complexes less susceptible to proteolytic attack in the GI tract. Partly elimination of tannins and phytic acid was observed after different thermal treatments (Table S1). The reduction of reactive tannins and phytic acid would result in less protein complexing and creating more space within the matrix, which increased the accessibility of the enzyme resulting in improved IVPD [11]. Trypsin inhibitors may interfere with the action of proteolytic enzymes in the GI tract by forming inactive complexes of trypsin and chymotrypsin. However, since trypsin inhibitors are heat-labile compounds, thermal processing should be an effective method for reducing its activity. Indeed, a marked decrease in trypsin inhibitors after different thermal processing was reported in many of the studies (Table S1) and a complete inactivation was also found for peas and kidney beans [23,25]. However, Embaby found that some treatments resulted in an increased level of anti-nutrients, but still obtained improved IVPD [26]. Thus, he suggested that ANFs are not solely responsible for lowering IVPD, and factors like cell wall rigidity and fiber content may influence the protein digestibility as such. 

In conclusion, the improvement of PD of legume proteins for all reported process methods is explained by the structural disintegration of the native protein concomitant with the reduction or even removal of anti-nutrients.

## 5. Challenges and Future Perspective 

The nutritional value of plant-based food is dependent on the amount of proteins, the specific distribution of the amino acids, and, most importantly, the bioavailability of these amino acids. Reduced protein quality will compromise the nutritional value, therefore maintaining quality and stability upon processing of plant-base materials is of utmost importance. From nature, proteins are not equally digestible, as their proteolytic susceptibility varies due to different three-dimensional structures according to their origin. This inherent difference in digestibility across legume sources is mirrored into the effect of processing methods. The benefits of food processing covers preservation, palatability, functional properties, or added convenience, but the impact of processing are often disconnected to the nutritional status of the proteins. Surely, the ultimate goals are plant-based products with high protein quality to be enjoyed as part of the consumer’s healthy diet and lifestyle. Assessing the quality of protein in relation to nutritional value means to determine the capacity of the proteins to satisfy the metabolic demand for amino acids. If perfect, a measure of the nutritional quality of dietary protein should provide the real protein digestibility and, thereby, enable the prediction of the overall efficiency of protein utilization. At present, various in vitro and in vivo protein digestibility methods exist, and neither of them can be used to accurately measure protein digestibility in absolute terms. An important conceptual difficulty is that proteins differ in their digestibility and the human GI tracts differ in their ability to digest proteins. Therefore, there is still much to learn and improve regarding methodologies for measuring and monitoring the protein digestibility before the “true” effect of processing on the important amino acids can be established. The golden goal for optimizing nutrition may be monitoring the individual protein digestive capacity in the individual body. However, even if food producers used the perfect PD method to optimize PD of a food product, the product must still appeal to consumers for success on the market. It is recognized that an increasing number of food companies are producing successful plant-based products providing variety, taste, and nutritional value to consumers.

## Supplementary Materials

The following are available online at https://www.mdpi.com/2304-8158/8/6/224/s1, Table S1. Protein digestibility (PD) of legumes using various processing methods.
