# Supplementary material for: The Effect of Processing on Digestion of Legume Proteins
Source: Foods. 2019 Jun 24;8(6):224. doi: 10.3390/foods8060224 (PMC6616939; doi:10.3390/foods8060224)
Supplement: Supplementary file 1 [file foods-08-00224-s001.pdf]

Table S1. Protein digestibility (PD) of legumes using various processing methods. ↓ indicates decreased PD. ↑ indicates increased PD. In addition, the effect on anti-nutritional factors (ANF) is given when provided in the references. Empty cell denotes that information was not provided.

Process	Type of legume (cultivar)	Specification	Soaked	Unsoaked	Process conditions (T; t; ΔH)	Methodology[1]	PD unprocessed (%)	PD processed (%)	Effect	Reference
Cooking	Bean flour (*Phaseolus vulgaris*)	*Raba*	*		30 min	CP	24.8	23.0	↓ (significantly)	[15]
		Warta	*		40 min		26.2	21.3		
	Pea (*Pisum sativum*)	*Milwa*	*		40 min		25.9	27.6	↑ (significantly)	
		Medal	*		40 min		28.4	27.5	↓ (significantly)	
	Lentil (*Lens culinaris*)	*Anita*	*		20 min		28.7	29.2		
		Tina	*		20 min		28.6	30.0	↑ (significantly)	
	Pea (*Pisum sativum L.)* flour	*Yellow-green (Windham)*	*		98 °C, 30 min	IVPD (trypsin, chymotrypsin, and peptidase, 10 min)	79.9	85.9	↑ (significantly)	[21]
		Yellow-green (Spector)	*		98 °C, 30 min		81.3	85.9		
		Green (Stirling)	*		98 °C, 30 min		82.0	85.9		
		Yellow (Fallon)	*		98 °C, 30 min		82.6	86.5		
		Pale green (Supra)	*		98 °C, 30 min		82.4	86.0		
		Yellow (Shawnee)	*		98 °C, 30 min		83.5	86.8		
		Green (Lifter)	*		98 °C, 30 min		83.5	86.3		
		Yellow-green (DSP)	*		98 °C, 30 min		82.3	86.8		
		Mean	*		98 °C, 30 min		82.2	86.3		

Full-fat soybean (FFSB)			*	80 °C, 1 min	SID (pig) of CP	46	52	↑ (Reduced trypsin inhibitors)	[16]
			*	100 °C, 6 min			73		
			*	100 °C, 16 min			80		
Pea (*Pisum sativum*) flour	*Green pea*	*		brought to a boil and maintained until done, 25–35 min	IVPD (trypsin, chymotrypsin and protease, 10 min)		86.56		[30]
	Yellow pea	*					86.75		
Lentil (*Lens culinaris*) flour	*Red lentil*	*		brought to a boil (100 °C) and maintained, 25–35 min	IVPD (trypsin, chymotrypsin and protease, 10 min)		84.67		[31]
	Green lentil	*					84.03		
Pea (*Pisum sativum L.*)			*	100 °C, 40 min—high heat; 30 min—simmered	TD	74.7	79.8	↑ (significantly)	[20]
Chickpea (*Cicer arietinum L.*)		*		100 °C, 90 min	IVPD (trypsin, pancreatin, 10 min)	83.61	88.52	↑ (significantly) (Reduced trypsin inhibitors, tannins, phytic acid)	[22]
Bitter lupin seed (*Lupinus termis*)			*	Cooked in distilled water (100 °C) 1:10 (w/v), 40 min	IVPD (multienzyme assay, 10 min)	78.55	80.73	↑ (significantly) (Reduced trypsin inhibitors, phytic acid (except sweet lupin), tannins)	[26]
		* (after cooking)					89.72		
Sweet lupin seed (*Lupinus albus*)			*			79.46	84.35		
		* (after cooking)					86.09		

	Vegetable pea (*Pisum sativum L.)*			*	Cooked in water (1:2 seed:water) at 100 °C	20 min	IVPD (trypsin, α-chymotrypsin, peptidase, 10 min)	73.5	76.0	↑ (Reduced trypsin inhibitors, phytic acid, tannins)	[23]
				*		30 min			77.2		
				*		40 min			78.3		
	Lentil (*Lens culinaris*)	*Pardina*		*	98 °C for 30 min		IVPD (trypsin, chymotrypsin, and peptidase)	79.1	83.6	↑ (significantly)	[24]
			*, 3h						83.3	↑	
			*, 12h						83.6		
		Crimson		*				79.4	82.2	↑ (significantly)	
			*, 3h						83.1		
			*, 12h						83.7		
	Chickpea (*Cicer arietinum L.*)			*				74.3	82.5	↑ (significantly)	
			*, 3h						82.9	↑ (significantly)	
			*, 12h						82.4	↑	
	Pea (*Pisum sativum*)	*Yellow*		*				82.0	84.0	↑ (significantly)	
			*, 3h						84.5	↑ (significantly)	
			*, 12h						84.3	↑	
		Green		*				82.6	84.1	↑ (significantly)	
			*, 3h						84.5	↑	
			*, 12h						83.6		
	Soybean (*Glycine max*)			*				71.8	83.8	↑ (significantly)	
			*, 3h						85.1		
			*, 12h						85.0		
	Black gram	*Punjab91*	*		Boiled, seed:water ratio of 1:5		IVPD (pepsin-HCl, 24 h)	34.8	65.0	↑ (significantly) (Reduced phytic acid, tannins)	[11]
	Chickpea	*CP-98*	*					36.0	69.0		
	Lentil	*Nayyab2002*	*					37.6	72.7		

	Kidney bean	*Red (Chkwal99)*	*				33.8	64.1		
		White (WK-70)	*				34.0	63.6		
	Kidney bean (*P. vulgaris L.*)	*Roba*		*	1:3 (w/v), 97 °C, 35 min	IVPD (multienzyme assay)	80.66	87.11	↑ (significantly) (Reduced trypsin inhibitors, phytic acid, tannins)	[25]
			*					90.31		
			$NaHCO_3$					90.34		
		Awash		*			71.14	78.28		
			*					78.98		
			$NaHCO_3$					78.88		
		Beshbesh		*			65.63	71.55		
			*					73.52		
			$NaHCO_3$					72.85		
Microwave cooking	Bean (*vicia faba var. major*)	*Windsor White*	*		500 J/g	IVPD (peptidase, trypsin, chymotrypsin)	46.0	57.1	↑ (significantly) (Reduced trypsin inhibitors)	[27]
			*		750 J/g			69.4		
			*		1000 J/g			76.5		
			*		1250 J/g			78.7		
			*		1500 J/g			79.0		
			*		1750 J/g			78.8		
			*		2000 J/g			78.8		
		Bacchus	*		500 J/g		52.2	68.0		
			*		750 J/g			73.2		
			*		1000 J/g			76.1		
			*		1250 J/g			78.4		
			*		1500 J/g			78.9		

			*		1750 J/g			79.0		
			*		2000 J/g			78.8		
			*		500 J/g			53.2		
			*		750 J/g			64.1		
			*		1000 J/g			78.2		
		Basta	*		1250 J/g		51.5	78.8		
			*		1500 J/g			79.8		
			*		1750 J/g			81.2		
			*		2000 J/g			81.0		
		Broad bean *(Vicia faba)*	*					77.4		
		Field bean *(Dolichos lablab)*	*					71.2		
	Bean	Green gram *(Phaseolus aureus Roxb)*	*					78.1		
		Horse gram *(Dolichos biflorus)*	*					69.8		
		French bean *(Phaseolus vulgaris)*	*					74.6		
	Lentil	*Lens esculenta*	*			IVPD (pepsin, pancreatin)		78.7		[13]
	Chickpea	*Bengal gram (Cicer arietinum)*	*					74.1		
	Pea	*Cowpea (Vigna catjang)*	*					73.7		

	Chickpea (*Cicer arietinum L.*)		*		on high level, 15 min		IVPD (trypsin, pancreatin, 10 min)	83.61	89.40	↑ (Reduced trypsin inhibitors, tannins, phytic acid)	[22]
	Bitter lupin seed (*Lupinus termis*)			*	seed:water ratio 1:10, for 6 min		IVPD (multienzyme method, 10 min)	78.55	80.54	↑ (significantly) (Reduced trypsin inhibitors, phytic acid, tannins)	[26]
			* (76 h after MW)						85.75		
	Sweet lupin seed (*Lupinus albus*)			*				79.46	80.67	↑ (significantly) (Reduced trypsin inhibitors, tannins)	
			* (24 h after MW)						81.51	↑ (Reduced trypsin inhibitors, tannins)	
	Vegetable pea (*Pisum sativum L.*)			*	2450 MHz	4 min	IVPD (trypsin, α-chymotrypsin, peptidase, 10 min)	73.5	74.2	↑ (Reduced trypsin inhibitors, tannins, and phytic acid (only at 12 min))	[23]
				*		8 min			75.1		
				*		12 min			75.5		
Pressure cooking	Moth bean (*Phaseolus aconitifolius Jacq.*)	*Local*		*	15 min		IVPD (pepsin, pancreatic)	70.3	78.1		[12]
			*		10 min				81.4		
		Jwala		*	15 min			71.9	78.9		
			*		10 min				81.9		

		RMO 225		*	15 min			72.3	78.2		
			*		10 min				83.9		
		RMO 257		*	15 min			74.7	82.4		
			*		10 min				85.2		
	Bean	Broad bean *(Vicia faba)*	*				IVPD (pepsin, pancreatin)		80.0		[13]
		Field bean *(Dolichos lablab)*	*						75.9		
		Green gram *(Phaseolus aureus Roxb)*	*						81.9		
		Horse gram *(Dolichos biflorus)*	*						78.4		
		French bean *(Phaseolus vulgaris)*	*						78.9		
	Lentil	*Lens esculenta*	*						82.6		
	Chickpea	Bengal gram *(Cicer arietinum)*	*						80.1		
	Pea	Cowpea *(Vigna catjang)*	*						80.5		
	Vegetable pea (*Pisum sativum L.*)			*	120 °C	10 min	IVPD (trypsin, α-chymotrypsin, peptidase, 10 min)	73.5	77.4	↑ (Reduced trypsin inhibitors, tannins, and phytic acid (only at 20 min))	[23]
				*		15 min			78.3		
				*		20 min			78.12		

Autoclaving	Bean flour (*Phaseolus vulgaris*)	*Raba*		*	121 °C, 7 min		CP	24.8	21.8	↓ (significantly)	[15]
		Warta		*	121 °C, 12 min			26.2	21.0		
	Pea flour (*Pisum sativum*)	*Milwa*		*	121 °C, 12 min			25.9	24.4		
		Medal		*	121 °C, 12 min			28.4	27.1		
	Lentil flour (*Lens culinaris*)	*Anita*		*	121 °C, 7 min			28.7	28.2	↓	
		Tina		*	121 °C, 7 min			28.6	28.7	↑	
	Yellow pea flour (*Pisum sativum, L.*)	Concentrate 1		*	108 °C, 8 min		AID and SID (chicken) of CP	AID 80.1	88.1	↑ (Reduced trypsin inhibitors)	[28]
								SID 88.7	95.0	↑ (significantly) (Reduced trypsin inhibitors)	
		Concentrate 2		*	108 °C, 8 min			AID 84.3	87.2	(Reduced trypsin inhibitors)	
								SID 91.6	95.0		
	Full-fat soybean (FFSB)			*	110 °C	15 min	SID (pig) of CP	46	82	↑ (Reduced trypsin inhibitors)	[16]
				*		30 min			83		
				*		45 min			84		
				*		60 min			82		
	Chickpea (*Cicer arietinum L.*)		*		121 °C, 35 min		IVPD (trypsin, pancreatin, 10 min)	83.61	89.96	↑ (Reduced trypsin inhibitors, tannins, phytic acid)	[22]

	Faba bean (*Vicia faba L.)*		*		120 °C, 20 min		IVPD (trypsin, chymotrypsin and peptidase, 10 min, protease, 9 min)	83.07	79.57	↓ (significantly)	[29]
	Lentil (*Lens culinaris Medikus*)		*					82.50	81.66	↓	
	Chickpea (*Cicer arietinum L.*)		*					78.28	83.16	↑ (significantly)	
	Dry white bean (*Phaseolus vulgaris L.*)		*					73.76	80.53	↑ (significantly)	
	Bitter lupin seed (*Lupinus termis*)			*	121 °C, 20 min		IVPD (multienzyme assay, 10 min)	78.55	82.84	↑ (significantly) (Reduced trypsin inhibitors, phytic acid (except sweet lupin), tannins)	[26]
			* (after treatment)						91.53		
	Sweet lupin seed (*Lupinus albus*)			*				79.46	83.93		
			* (after treatment)						85.95		
	Black gram	*Punjab91*	*		121 °C	10 min	IVPD (pepsin-HCl, 24h)	34.8	68.0		[11]
						20 min			64.5		
						40 min			62.8		
						60 min			62.0		
						90 min			61.8		

					128 °C	20 min			62.4			
	Chickpea	*CP-98*	*		121 °C	10 min		36.0	72.5			
						20 min			68.5			
						40 min			67.0			
						60 min			65.7			
						90 min			64.9	↑ (significantly) (Reduced phytic acid, tannins)		
					128 °C	20 min			65.0			
	Lentil	*Nayyab2002*	*		121 °C	10 min		37.6	76.0			
						20 min			72.0			
						40 min			70.8			
						60 min			68.0			
						90 min			67.8			
					128 °C	20 min			68.0			
	Kidney bean	*Red (Chkwal99)*	*		121 °C	10 min		33.8	68.3			
						20 min			64.0			
						40 min			62.4			
						60 min			61.0			
						90 min			60.5			

					128 °C	20 min			61.8		
		White (WK-70)	*		121 °C	10 min		34.0	69.8		
						20 min			63.0		
						40 min			61.4		
						60 min			60.0		
						90 min			59.9		
					128 °C	20 min			61.0		
	Kidney bean (*P. vulgaris L.*)	*Roba*		*	1:3 (w/v), 121 °C, 30 min		IVPD (multienzyme assay)	80.66	86.31	↑ (significantly) (Reduced trypsin inhibitors, phytic acid, tannins)	[25]
			*						92.76		
			$NaHCO_3$						92.84		
		Awash		*				71.14	76.12		
			*						82.53		
			$NaHCO_3$						81.82		
		Beshbesh		*				65.63	69.58		
			*						74.77		
			$NaHCO_3$						74.83		
Baking	Dry pea flour (*Pisum sativum*)	*Yellow pea*		*	198.3 °C, 198.3 °C, and 165.6 °C	35 min	IVPD (trypsin, chymotrypsin, and protease, 10 min)		82.49		[30]
		Green pea		*		29 min			82.22		
		Red lentil		*			IVPD		85.03		[31]

	Lentil flour (*Lens culinaris*)	*Green lentil*		*	198.3 °C, 198.3 °C, and 165.6 °C	35 min	(trypsin, chymotrypsin and protease, 10 min)		79.33		
Extrusion	Dry pea flour (*Pisum sativum*)	*Yellow pea*		*	30–50 °C, 70–90 °C, and 100–120 °C		IVPD (trypsin, chymotrypsin and protease, 10 min)		86.93		[30]
		Green pea		*					90.0		
	Lentil flour (Lens culinaris)	*Red lentil*		*	30–50 °C, 70–90 °C, and 100–120 °C		IVPD (trypsin, chymotrypsin and protease, 10 min)		88.01		[31]
		Green lentil		*					84.30		
	Pea (*Pisum sativum L., Tarachalska cv.*)			*	135 ± 10 °C		AID of CP	74.3	85.9	↑ (significantly) (Reduced trypsin inhibitors, phytic acid)	[34]
	Common Bean flour (*Phaseolus vulgaris, L.*)	*BRS pontal (carioca)*		*	150 °C		IVPD (pepsin, pancreatin)	28.16	48.52	↑ (significantly) (Reduced trypsin inhibitors, phytic acid)	[14]
		BRS grafite (black)		*				28.62	52.80		
	Pea flour (*Pisum sativum L.*)			*	145 °C		IVPD (trypsin,α-chymotrypsin, peptidase)	839 g/kg	874 g/kg	↑ (significantly) (Reduced trypsin inhibitors, phytic acid, tannins, polypehnols)	[33]
	Kidney Bean flour (*Phaseolus*	*100 g/kg of extruded*		*	150 °C		AID of CP	89.60 (without kidney	85.50	↑ (significantly)	[35]

	vulgaris L. var. Pinto)	*kidney bean (EKB)*					bean), 79.03 (100 g/kg raw KB), 79.53 (200 g/kg raw KB), 77.23 (300 g/kg raw KB)			
		200 g/kg of EKB		*				85.50		
		300 g/kg of EKB		*				85.90		
	Bean flour	Common bean (*Phaseolus vulgaris, L.)*		*	152 °C, 156 °C	IVPD (trypsin,α-chymotrypsin, peptidase, 10 min)	68.1	83.0	↑ (significantly) (Reduced trypsin inhibitors, phytic acid, tannins, polypehnols)	[32]
		Broad bean (*Vicia faba)*		*			70.8	87.4		
New processing methods	Lentil (*Lens culinaris*)	*Pardina*	*[1], 1.5 h		47 kHz	IVPD (trypsin, chymotrypsin, and peptidase)	79.1	79.8	↑ (significantly)	[24]
			*[1], 3 h					80.1		
			*[2], 0.5 h		621 MPa			79.1		
			*[2], 1 h					79.7	↑ (significantly)	
		Crimson	*[1], 1.5 h		47 kHz		79.4	80.2	↑ (significantly)	
			*[1], 3 h					80.9		
			*[2], 0.5 h		621 MPa			79.8		
			*[2], 1 h					81.0	↑ (significantly)	
	Chickpea (*Cicer arietinum L.*)		*[1], 1.5 h		47 kHz		74.3	74.1		
			*[1], 3 h					74.5		
			*[2], 0.5 h		621 MPa			74.9	↑ (significantly)	
			*[2], 1 h					76.2		
	Pea (*Pisum sativum*)	*Yellow*	*[1], 1.5 h		47 kHz		82.0	81.2	↓ (significantly)	
			*[1], 3 h					81.2		
			*[2], 0.5 h		621 MPa			81.2		
			*[2], 1 h					81.3		
		Green	*[1], 1.5 h		47 kHz		82.6	81.9	↓ (significantly	
			*[1], 3 h					82.0		

			*[2], 0.5 h		621 MPa			81.2		
			*[2], 1 h					82.0		
	Soybean (*Glycine max*)		*[1], 1.5 h		47 kHz		71.8	72.1	↑	
			*[1], 3 h					71.6	↓	
			*[2], 0.5 h		621 MPa			72.2	↑ (significantly)	
			*[2], 1 h					73.1		
	Dry split pea (*Pisum sativum*)				350 MPa, 45 min, 40 °C	IVPD (trypsin)	82.3	80.7 (mean of all three)	↓ (Reduced trypsin inhibitors, phytic acid)	[10]
					600 MPa, 60 min, 60 °C			85.8	↑ (Reduced trypsin inhibitors, phytic acid)	
					100 MPa, 60 min, 20 °C			78.6	↓ (Reduced trypsin inhibitors, phytic acid)	
					600 MPa, 30 min, 20 °C			82.3	(Reduced trypsin inhibitors, phytic acid)	
					100 MPa, 60 min, 60 °C			79.2	↓ (Reduced trypsin inhibitors, phytic acid)	
					600 MPa, 30 min, 60 °C			83.3	↑ (Reduced trypsin inhibitors, phytic acid)	
					100 MPa, 30 min, 60 °C			79.6	↓	

					600 MPa,<br>60 min, 20 °C			82.1	(Reduced trypsin inhibitors, phytic acid)	
					100 MPa,<br>30 min, 20 °C			79.9		
	White beans (*Phaseolus vulgaris*)				350 MPa,<br>45 min, 40 °C		69.1	68.0 (mean of all three)	↓ (Reduced trypsin inhibitors, phytic acid)	
					600 MPa,<br>60 min, 60 °C			75.1	↑ (Reduced trypsin inhibitors, phytic acid)	
					100 MPa,<br>60 min, 20 °C			68.5	↓ (Reduced trypsin inhibitors, phytic acid)	
					600 MPa,<br>30 min, 20 °C			69.0		
					100 MPa,<br>60 min, 60 °C			67.5		
					600 MPa,<br>30 min, 60 °C			68.8		
					100 MPa,<br>30 min, 60 °C			68.8		
					600 MPa,<br>60 min, 20 °C			68.9		
					100 MPa,<br>30 min, 20 °C			63.8		

[1] CP: Srude protein, IVPD: *In vitro* protein digestibility, SID: standardized ileal digestibility, TD: True digestibility, AID: Apparent ileal digestibility

*[1] Soaked under ultrasound, *[2] Soaked under high pressure.
